# Pharmacological basis for medicinal use of *Cymbopogon proximus* Hochst. ex A. Rich. Essential oil in hyperactive gastrointestinal disorders

**DOI:** 10.3389/fphar.2025.1533511

**Published:** 2025-06-30

**Authors:** Hassan N. Althurwi, Najeeb Ur Rehman, Maged S. Abdel-Kader, Faisal F. Albaqami

**Affiliations:** ^1^ Department of Pharmacology and Toxicology, College of Pharmacy, Prince Sattam bin Abdulaziz University, Al-Kharj, Saudi Arabia; ^2^ Pharmacognosy Department, College of Pharmacy, Prince Sattam bin Abdulaziz University, Al-Kharj, Saudi Arabia; ^3^ Department of Pharmacognosy, College of Pharmacy, Alexandria University, Alexandria, Egypt

**Keywords:** *Cymbopogon proximus*, antidiarrheal, isolated ileum, antimuscarinic, calcium channel blocker

## Abstract

**Background:**

Cymbopogon is used in traditional medicine in tropical and subtropical regions to treat various ailments. However, no research has yet provided a detailed pharmacodynamic explanation for its antidiarrheal and antispasmodic effects. This study aimed to evaluate the antidiarrheal and antispasmodic properties of the essential oil of *Cymbopogon proximus* (EOCP), with the goal of scientifically validating its traditional use in folk medicine.

**Methods:**

An animal study of antidiarrheal activity was carried out using a castor oil-induced diarrhea model in rats, and the isolated small intestine of rats was used to investigate the specific mechanisms of the antispasmodic effects.

**Results:**

The EOCP, exhibited a protective effect against castor oil-induced diarrhea in rats at 100 and 200 mg/kg, similar to the standard drug, dicyclomine. In rat ileum preparations, EOCP decreased the basal tonus with a maximal response of (R_max_; % of ACh.-contraction) of 18.5% ± 1.5% compared with 17.5% ± 2.5% achieved with dicyclomine. In contractions elicited by different agents, EOCP showed higher potency in relaxing carbachol (CCh)-induced contractions compared to those induced by high K^+^ concentrations. It showed a similar relaxation profile to dicyclomine but differed from the inhibitory effects of verapamil and/or atropine. Pre-incubation of isolated ileum with increasing doses of EOCP showed that at a lower concentration (0.03 mg/mL), EOCP induced a rightward parallel shift in carbachol (CCh) concentration-response curves. At a higher concentration (0.1 mg/mL), it caused a non-parallel shift with a reduction in the maximum response, similar to the effects of dicyclomine, a dual inhibitor of muscarinic receptors and Ca^++^ channels. The Ca^++^ channel inhibitory effect of EOCP was confirmed by its rightward shift of Ca^++^ concentration-response curves and suppression of the maximal response, resembling the effects of verapamil, a well-established Ca^++^ antagonist.

**Conclusion:**

These results suggest that EOCP produces antidiarrheal and antispasmodic effects, possibly mediated via direct effect on intestinal smooth musclesf followed by dual inhibition of muscarinic receptors and Ca^++^ channels, thus providing a pharmacological basis for its traditional use in hyperactive gut disorders such as diarrhea and spasms.

## 1 Introduction

*Cymbopogon,* known for its rich essential oil content, has long been used in traditional medicine across the tropical and subtropical regions of Asia, Africa, and the Americas (Avoseh et al., 2015). Its medicinal uses include treatment of ailments such as cough, fever, infections, cancer, and digestive disorders (Dutta et al., 2016). Cymbopogon lowers blood pressure in normotensive rats and provides protection against L-NAME-induced hypertension (El-Nezhawy et al., 2014; Althurwi et al., 2020). Laboratory and animal studies demonstrate its pharmacological benefits, including anticancer, heart-protective, cholesterol-lowering, antioxidant, anti-inflammatory, anti-diabetic, and antimicrobial effects (Mansour et al., 2002; Adeneye and Agbaje, 2007; Miguel, 2010; Selim, 2011; Ekpenyong et al., 2014).

*Cymbopogon proximus* (commonly known as Halfabar or Maharaib) is a highly aromatic grass commonly found in Southern Egypt and Northern Sudan*. C. proximus* is traditionally used as a diuretic and antispasmodic, which is attributed to its potent smooth muscle-relaxing effects (El-Askary et al., 2003). Additionally, it possesses various biological activities, including hypoglycemic, antipyretic, bronchodilatory, antibacterial, anticonvulsant, and antiemetic properties (El-Askary et al., 2003; Selim, 2011; Ibrahim and El-Khateeb, 2013; El-Nezhawy et al., 2014; Warrag et al., 2014). In our earlier conducted study on the essential oil of this plant (Althurwi et al., 2020), the detailed phytochemical *GC-MS* analysis revealed the presence of elemol, piperitone, α-eudesmol and β-eudesmol in as the plant major constituents. Interestingly, some of the identified components in *C. proximus* are known to have spasmolytic properties such as, piperitone, with previously reported findings of having concentration-dependent spasmolytic activity (Ponce-Monter et al., 2008). Moreover, β-eudesmol, found in *C. Proximus* essential oil, has been explored for its relaxing effect in previous studies (Morita et al., 1996). Although its antispasmodic and smooth muscle relaxant effects have been established in previous studies (Abdel-Moneim et al., 1969), no detailed pharmacodynamic explanation for its antidiarrheal and antispasmodic effects has been provided. Therefore, the current study aimed to scientifically investigate the antidiarrheal and antispasmodic properties of the essential oil of *Cymbopogon proximus* (EOCP), to provide a pharmacological basis for its traditional use in the treatment of hyperactive gut disorders like diarrhea and spasms. While *Cymbopogon proximus* has long been used in traditional medicine, no previous research has provided a clear mechanistic explanation for its effectiveness in treating hyperactive gut disorders. This study identifies EOCP’s dual inhibitory effect on muscarinic receptors and calcium channels and positions it as a natural alternative to established pharmaceuticals such as dicyclomine. The broader significance of these findings extends to the potential development of EOCP as a plant-based therapeutic for gastrointestinal conditions, offering a safer, natural remedy at a time of increasing interest in alternative and complementary medicine. Additionally, this research could open avenues for further exploration of other *Cymbopogon* species and their medicinal properties, thereby contributing to the advancement of ethnopharmacology and drug development from natural products.

## 2 Materials and methods

### 2.1 Plant material and extraction

*Cymbopogon proximus* belonging to the Poaceae family, was sourced from a local market in Alexandria, Egypt. The plant’s identity was confirmed by Prof. Saniya Kamal of the Department of Botany, College of Science, Alexandria University, Alexandria, Egypt.

### 2.2 Extraction of the essential oil Cymbopogon proximus

Essential oil was extracted from 250 g of dried, powdered *C. proximus* by hydrodistillation for 5 h of (Meyer-Warnod, 1984). The oil was then separated and dried with anhydrous sodium sulfate, giving a final yield of 5.4% w/w (Althurwi et al., 2020).

### 2.3 Chemicals

Acetylcholine chloride (ACh), atropine, carbachol (CCh), dicyclomine, Tween 80 and verapamil were sourced from Sigma Chemicals Co. (St. Louis, MO, United States). The physiological salt solutions were prepared using potassium chloride (Sigma Chemicals Co.), calcium chloride, magnesium chloride, magnesium sulfate, glucose, sodium bicarbonate, potassium dihydrogen phosphate, and sodium dihydrogen phosphate (Merck, Darmstadt, Germany), ethylenediaminetetraacetic acid (EDTA), and sodium chloride (BDH Laboratory Supplies, Poole, England). All chemicals used were of analytical grade. Castor oil was obtained from a local pharmacy in Al-Kharj. EOCP was administered in a volume of 10 mL/kg after dissolving in a vehicle of 1% (w/v) Tween 80 in saline in the *in-vivo* assay whereas for the *ex-vivo* studies, EOCP stock solution and dilutions were prepared in Tyrode’s solution. The prepared stock solutions of EOCP were sonicated just before use.

### 2.4 Animals

Male Wistar albino rats, weighing 200–250 g, were obtained from the Lab Animal Care Unit of the College of Pharmacy, Prince Sattam Bin Abdulaziz University (Al-Kharj, KSA). The animals were housed under a 12-h light/dark cycle and received food and water *ad libitum.* After a 24-h fasting period, the rats were humanely euthanized by a blow to the head, followed by cervical dislocation. All experiments were conducted in accordance with the guidelines set by the Institute of Laboratory Animal Resources, Commission on Life Sciences, National Research Council (1996). The assay protocol was approved by the Standing Committee of Bioethics Research (SCBR) at Prince Sattam Bin Abdulaziz University with reference number SCBR-394/2024.

### 2.5 Castor oil-induced diarrhea

Rats were subjected to 24 h fasting and kept individually in separate cages with saw dust replaced with a clean blotting sheet. They were divided into five groups, each consisting of five rats. Each rat in the first group received saline (1% Tween 80 at 10 mL/kg, orally) and was recorded as a negative control. Effective doses of plant oil were previously determined on a trial basis, with the second and third groups receiving two increasing doses of EOCP as 100 and 200 mg/kg previously dissolved in vehicle (1% Tween 80 at 10 mL/kg, orally). The fourth and fifth groups received the positive control drug, dicyclomine, at oral doses of 50 and 100 mg/kg, respectively. Each rat was carefully subjected to 10 mL/kg dose of castor oil after approximately 1 h of sample treatment. After 4 h of the castor oil dosing, the cages were subsequently monitored for loose spots of diarrhea, and their absence was recorded as a positive result, indicating protection from diarrhea (Rehman et al., 2012).

### 2.6 Rat ileum

The rat’s abdomen was opened, and with due care, the ileum was isolated by tracing it from the ileo-cecal junction, placed in a normal buffer (Tyrode’s solution), and mesenteries were removed (Rehman et al., 2024). A 2-cm segment of the ileum was suspended in a 10 mL tissue bath filled with Tyrode’s solution (pH 7.4), maintained at 37°C, and aerated with a mixture of 95% O_2_ and 5% CO_2_ (carbogen). The composition of the Tyrode’s solution (in mM) was as follows: NaCl 136.9, KCl 2.7, MgCl_2_·6H_2_O 0.5, NaHCO_3_ 11.9, NaH_2_PO_4_·2H_2_O 0.32, CaCl_2_ 1.8, and glucose 5.05. One end of the ileum was secured to a metal tissue hook, while the other was attached via a cotton thread to an isotonic transducer connected to an emkaBath (France) system. Tissue responses were recorded using IOX software (version 2.9.10.6, emka technologies, SAS, Paris, France). Each tissue segment was subjected to an initial load of 1 g and allowed to equilibrate for 30 min before drugs were administered. After the equilibration phase, each tissue preparation was stabilized using a submaximal concentration of ACh, (0.3 μM) at 3-min intervals, until stable responses were observed. The inhibitory effect of the test substances was evaluated against contractions induced by CCh (1 μM) and high potassium (80 mM). Carbachol, a muscarinic receptor agonist, induces strong contractions in isolated ileum tissue, and any substance that reverses these contractions is considered anti-muscarinic (Arunlakshana and Schild, 1997).

To confirm whether the inhibitory substance acts competitively or non-competitively on muscarinic receptors, control concentration-response-curves (CRCs) for CCh-were generated by incrementally increasing the CCh concentration. CRCs for CCh were repeated with increasing concentrations of the test substance (EOCP) and control drugs (dicyclomine, verapamil, and atropine), as described by Choo and Mitchelson (1978).

For determining the calcium channel-blocking signaling, high potassium (80-mM) was used to contract the tissue sample, as outlined by Farre et al. (1991). Potassium concentrations (>30 mM) have been reported to cause smooth muscle depolarization by activating membrane voltage-dependent calcium signaling channels, thus facilitating the entry of extracellular calcium and triggering contractions. A substance that inhibits these contractions is considered a blocker of calcium influx through L-type calcium channels (Godfraind et al., 1986). Once the contraction reached a plateau (usually within 7–10 min), the test material was added cumulatively to achieve concentration-dependent inhibitory effects.

To further verify the calcium antagonist activity of the test substance, the tissue was first stabilized in normal Tyrode’s solution. This was then replaced with a calcium-free Tyrode’s solution containing 0.1 mM EDTA for 30 min to remove calcium from the tissue. The solution was then exchanged for a potassium-rich, calcium-free Tyrode’s solution with the following composition (in mM): NaCl 91.03, KCl 50, MgCl_2_·6H2O 0.50, NaHCO_3_ 11.9, NaH_2_PO_4_·2H_2_O 0.32, glucose 5.05, and EDTA-Na_2_·2H_2_O 0.1. After incubating for 30 min, control calcium concentration-response curves (CRCs) were generated. Once the calcium CRCs had stabilized (typically after two cycles), the tissue was exposed to the test compound for 1 h. Calcium CRCs were then reconstructed at varying concentrations of the test substance to evaluate their calcium antagonist effect.

### 2.7 Statistical analyses

Data are expressed as mean ± standard error of the mean (SEM, n = number of experiments), along with median effective concentrations (EC_50_) and corresponding 95% confidence intervals (CI). Statistical analysis for the antidiarrheal assay was performed using the Chi-square test, while potency of the agonist control CRCs) was compared with its potency in the presence of the different antagonist(s) using Oneway ANOVA and Dunnett’s multiple comparison test. Repeated Measures ANOVA followed by Bonferroni’s post-test for comparisons of CRCs of Ca^++^ with their respective controls. Differences were considered statistically significant at P < 0.05.

Concentration-response curves were evaluated using non-linear regression analysis with GraphPad software (GraphPAD, San Diego, CA, United States).

## 3 Results

### 3.1 Effect on castor oil-induced diarrhea

The tested oil (EOCP) exhibited dose-dependent protective effects (40% and 80%) against castor oil-induced diarrhea in rats at 100 and 200 mg/kg, respectively. All rats of the negative control group (saline treated) were recorded with loose diarrheal spots and therefore did not exhibit protection against castor oil-induced diarrhea. However, the positive control drug, dicyclomine, showed 40% protection at a lower dose of 50 mg/kg, while its higher dose (100 mg/kg) showed complete (100%) protection (Table 1).

**TABLE 1:** Antidiarrheal activity of the essential oil of *Cymbopogon proximus* (EOCP) in rats with diarrhea induced castor oil (10 mL/kg).

Treatment (p.o.), dose (mg/kg)	No. of rats out of five with diarrhea	% Protection
Saline (10 mL/kg) + castor oil	5/5	0
EOCP + castor oil		
100 + 10 200 + 10	3^a^/5 1^a^/5	40 80
Dicyclomine + castor oil		
50 + 10 100 + 10	3^a^/5 0^b^/5	40 100

^a^ *p* <0.05 and.

^b^ *p* <0.01 vs. saline + castor oil treated group (χ2-test).

### 3.2 Relaxant effect on the baseline of rat ileum

**FIGURE 1:**
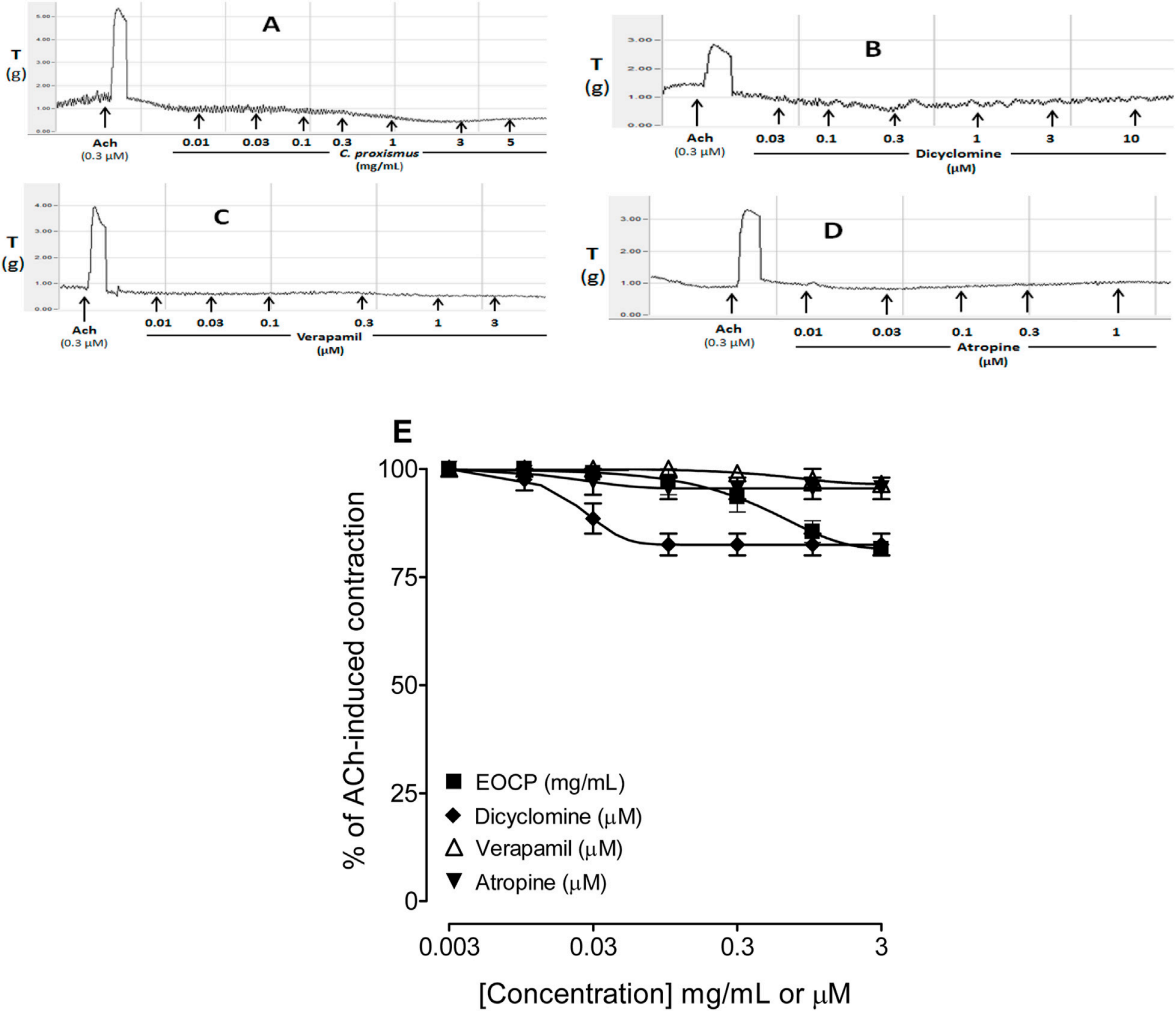
Original recordings to observe any stimulant and/or inhibitory effect on the baseline contractions of isolated rat ileum preparations by the increasing concentration of **(A)** essential oil of *Cymbopogon proximus* (EOCP), **(B)** dicyclomine, **(C)** verapamil, and **(D)** atropine at approximately 2–4 min intervals. The % inhibitory baseline effect was calculated by considering ACh (0.3 µM) contraction as 100%. compared with acetylcholine (Ach; 0.3 µM)-induced contraction **(E)**. Data are presented as mean ± SEM, n = 6.

Figures 1A–D shows the original representative tracings of the effect of EOCP, dicyclomine, verapamil and atropine on the baseline contraction of the rat isolated ileum. The essential oil of *C. proxismus* was shown to have a direct relaxant effect on the baseline intestinal tone (Figure 1A) with a maximum relaxant response (R_max_) corresponded to 18.5% ± 1.5% of the ACh (0.3 µM)-induced contraction (Figure 1E). This effect of EOCP was equivalent to the maximal relaxant response induced by dicyclomine (Figure 1B) which was recorded with R_max_ of 17.5% ± 2.5% (Figure 1E) whereas verapamil (Figure 1C) and atropine (Figure 1D) relaxed only slightly the baseline contraction of ileum tissue with resultant R_max_ of 3.5% ± 1.5% and 4.5% ± 2.5%, respectively (Figure 1E).

### 3.3 Antispasmodic effect on the induced contractoins of rat ileum

**FIGURE 2:**
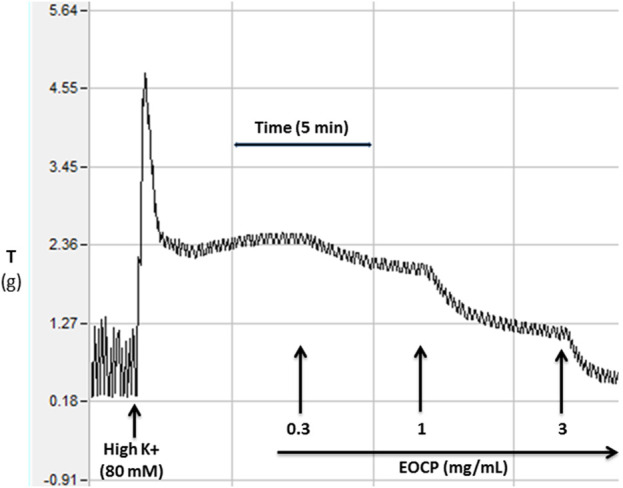
Original recordings showing induced contractions in isolated rat ileum by high K^+^ (80 mM) and the inhibitory effect of the essential oil of *Cymbopogon proximus* (EOCP). The time interval between two concentrations was approximately 5–10 min whereas Y-axis showing the tissue tension (T) in grams (g).

**FIGURE 3:**
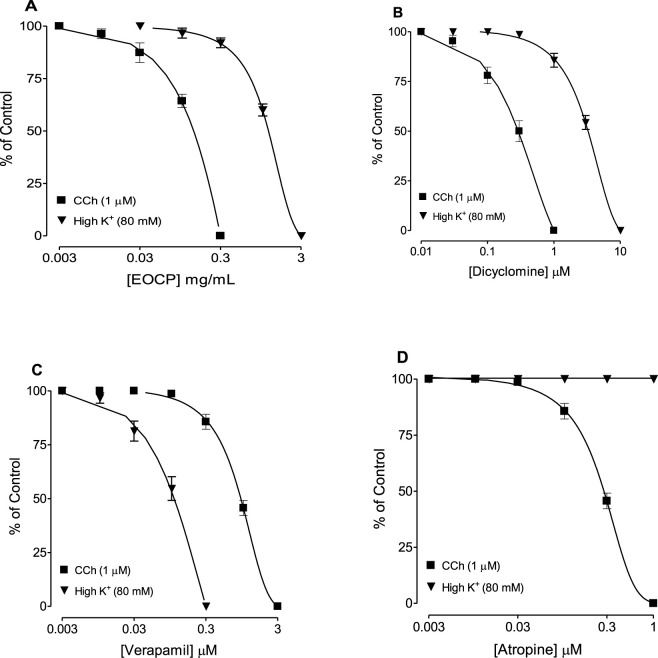
Concentration-response curves comparing the inhibitory effects of **(A)** essential oil of *Cymbopogon proximus* (EOCP), **(B)** dicyclomine, **(C)** verapamil, and **(D)** atropine on carbachol (CCh) and high K^+^-induced contractions in isolated rat ileum preparations. Data are presented as mean ± SEM, n = 5.

Figure 2 shows the representative concentration-dependent antispasmodic effect of the EOCP on high K^+^-induced contractions. In both, CCh (1 µM) and K^+^ (80 mM)-induced contractions, complete relaxation was observed by EOCP. However, EOCP reversed CCh-induced contractions at lower concentrations, with a recorded EC_50_ of 0.12 mg/mL (0.10–0.15), n = 5, demonstrating higher potency compared to its inhibitory effect against K^+^-induced contractions where lower potency was observed with resultant EC_50_ of 1.15 mg/mL (1.07–1.28), n = 5, as shown in Table 2 and Figure 3A. Dicyclomine, also showed a similar pattern of inhibitory activity against CCh- and K^+^-induced contractions with corresponding EC_50_ values of 0.31 μM (0.24–0.41), n = 5 and 3.41 μM (3.01–3.86), n = 5 (Figure 3B). However, verapamil exhibited greater potency against K^+^-induced contractions, with EC_50_ of 0.11 μM (0.08–0.13), n = 5, and its effect on CCh-induced contractions, had an EC_50_ 0.92 μM (0.83–1.01), n = 5, Figure 3C. Control drug, atropine effectively counteracted contractions induced by CCh (1 µM) with an EC_50_ of 0.29 μM (0.26–0.32), n = 5, without any effect on K^+^-induced contractions (Figure 3D). The detailed EC_50_ values for EOCP and control drugs are shown in Table 2.

**TABLE 2:** Comparison of EC_50_ values for the inhibitory effect of the essential oil of *Cymbopogon proximus* (EOCP) and positive control drugs (dicyclomine, atropine, and verapamil) on carbachol (CCh, 1 µM) and high K^+^ (80 mM)-induced contractions in isolated rat ileum.

Parameters	EOCP	Dicyclomine	Atropine	Verapamil
CCh	0.12 mg/mL (0.10–0.15), n = 5	0.31 μM (0.24–0.41), n = 5	0.29 μM (0.26–0.32), n = 5	0.92 μM (0.83–1.01), n = 5
High K^+^	1.15 mg/mL (1.07–1.28), n = 5	3.41 μM (3.01–3.86), n = 5	0% inhibition, n = 5	0.11 μM (0.08–0.13), n = 5

### 3.4 Anticholinergic activity confirmation

**FIGURE 4:**
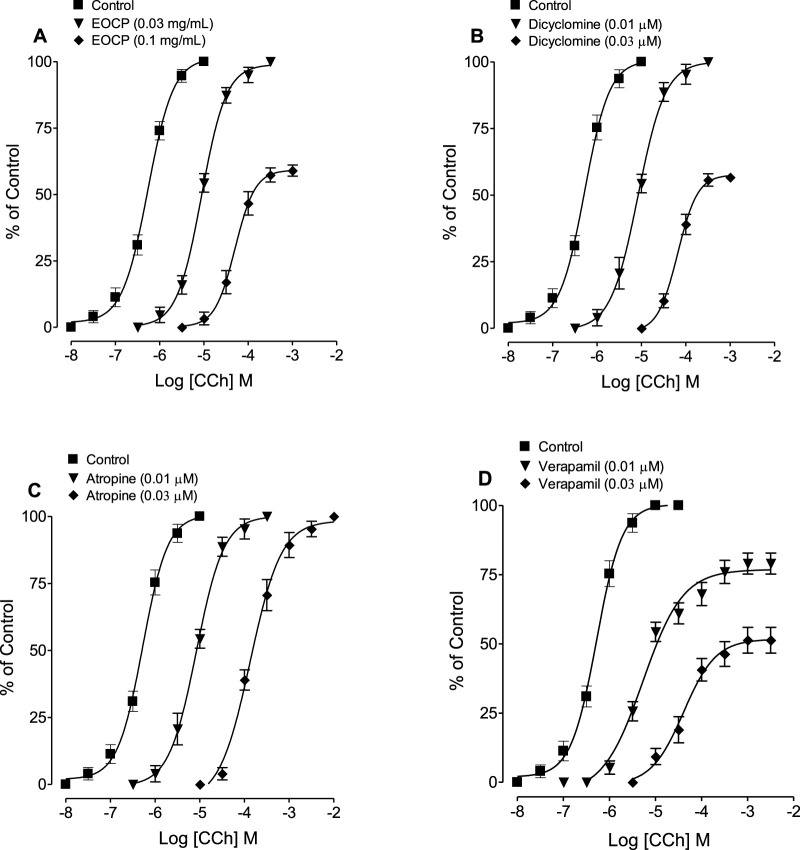
Concentration-response curves of carbachol (CCh) in the absence and presence of different concentrations of **(A)** the essential oil of *Cymbopogon proximus* (EOCP), **(B)** dicyclomine, **(C)** atropine, and **(D)** verapamil in isolated rat ileum preparations. Data are presented as mean ± SEM, n = 5-6.

At a lower concentration of 0.03 mg/mL, EOCP caused a rightward parallel shift in the CCh-CRCs without reducing the maximum contractile response of the agonist, followed by a non-parallel shift with suppression of the maximal response at a higher concentration of 0.1 mg/mL (Figure 4A; Table 3). Similarly, dicyclomine (0.01 and 0.03 µM) showed a similar shift pattern by showing suppression of the maximum response only at higher concentration of 0.03 µM where the maximum contractile response of CCh was recorded as 56.6% whereas its lower concentration significantly effected CCh potency (p < 0.01) without decreasing the maximum response of CCh (Table 3; Figure 4B). Pre-incubation with atropine (0.01 and 0.03 µM) caused a rightward parallel shift in the CCh-CRCs without suppressing the maximal effect while the potency of CCh was significantly effected (p < 0.01) at both concentrations (Table 3; Figure 4C). In contrast, verapamil (0.01 and 0.03 µM) caused a non-parallel rightward shift in CCh-CRCs with a decrease in the maximum response of control curves to 79% and 51.3% at verapamil pre-incubated concentration of 0.01 µM and 0.03 µM, respectively (Table 3; Figure 4D).

**TABLE 3:** Effects of different inhibitors/antagonists (EOCP, atropine, dicyclomine, verapamil) on potency of carbachol (CCh) at producing contractile effect in rat isolated ileum preparations, expressed as EC_50_ values.

CCh potency (-Log M)	Complete emax. With EC_50_	Maximum % stimulant effect (mean ± SEM)
Control	0.54 µM (0.45–0.64), n = 6	
EOCP 0.03 mg/mL	8.89 µM (7.62–10.38)**, n = 6	
EOCP 1.00 mg/mL		59.0% ± 2.08%, n = 5
Control	0.52 µM (0.43–0.63), n = 6	
Atropine 1 × 10^−8^ M	8.42 µM (6.78–10.60)**, n = 5	
Atropine 3 × 10^−8^ M	135.6 µM (102.6–179.1)**, n = 6	
Control	0.52 µM (0.43–0.63), n = 6	
Dicyclomine 1 × 10^−8^ M	8.42 µM (6.78–10.46)**, n = 5	
Dicyclomine 3 × 10^−8^ M		56.6% ± 1.66%, n = 5
Control	0.52 µM (0.44–0.61), n = 6	
Verapamil 1 × 10^−8^ M		79.0% ± 3.78%, n = 6
Verapamil 3 × 10^−8^ M		51.3% ± 6.66%, n = 5

Values represent geometric means along with 95% confidence intervals in parenthesis. In preparation, where complete inhibition was not achieved, the data were presented as the maximum stimulant response for comparison. “n” represents number of observations. Asterisks denote significance of difference between CCh, potency in the absence (respective controls) to its potency in the presence of the different concentrations of the essential oil (EOCP), atropine, dicyclomine and verapamil (Oneway ANOVA, and Dunnett’s multiple comparison test: **p < 0.01).

### 3.5 Ca^++^ channel blocking activity confirmation

**FIGURE 5:**
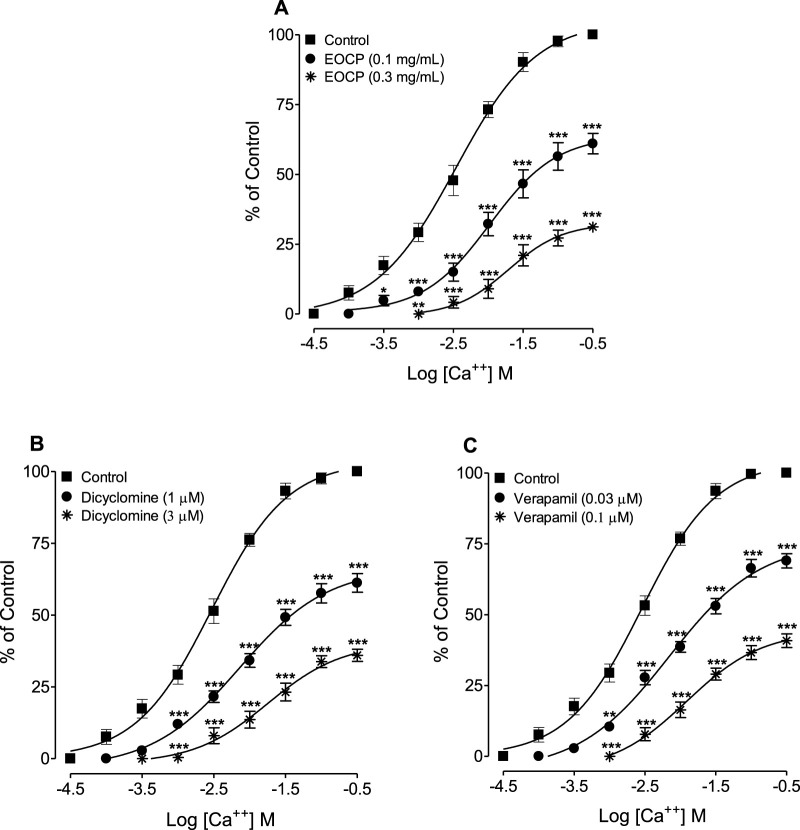
Concentration-response curves of Ca^++^ in the absence and presence of different concentrations of **(A)** the essential oil of *Cymbopogon proximus* (EOCP), **(B)** dicyclomine, and **(C)** verapamil in isolated rat ileum preparations. Data are presented as mean ± SEM, n = 5. **p < 0.01, and ***p < 0.001 shows comparison of the mean of Ca^++^-mediated contractions in the pretreated tissues with EOCP, dicyclomine and verapamil with the respective mean of Ca^++^-mediated contractions in control (untreated) ileum preparations (Repeated Measures ANOVA, followed by Bonferroni post-test).

When tested for possible interactions with Ca^++^ channels, EOCP (0.1 and 0.3 mg/mL) induced a rightward shift of Ca^++^ concentration-response curves (CRCs) and suppressed the maximal response (Figure 5A), mirroring the effect of verapamil (Figure 5B) and dicyclomine (Figure 5C).

## 4 Discussion

Given the known medicinal use of *C. proximus* as an antispasmodic (El-Askary et al., 2003) and its smooth muscle relaxant properties (Abdel-Moneim et al., 1969), the extracted essential oil was tested on laboratory rats to evaluate its potential effects on hyperactive gut disorders, such as diarrhea and spasms. In a castor oil-induced diarrhea model, EOCP demonstrated a dose-dependent protective effect similar to dicyclomine, a standard antidiarrheal agent (Pasricha, 2006). Castor oil causes diarrhea due to the ricinoleic acid, formed during hydrolysis, which disrupts electrolyte and water transport, and leads to strong intestinal contractions (Iwao and Terada, 1962; Croci et al., 1997). Therefore, an effective antidiarrheal remedy can work by inhibiting bowel contractions.

Essential oil of *C. proxismus* had a direct relaxant effect on the isolated ileum preparation of rat and also relaxed tissue with induced contractions by different spasmodic agents. These effects were observed at lower concentrations and might be related to the symptomatic relief of the acute abdominal pain associated with some gastric disturbances (Câmara et al., 2003). Next, to explore the mechanism underlying EOCP’s antidiarrheal effects, its activity was tested against contractions induced by CCh and high potassium (K^+^). Medicinal plants extracts or thier extracted essential oils usually possess antispasmodic effect on multiple smooth muscles including intestinal smooth muscles, possibly mediated by combination of mechanism(s) such as anticholinergic (Mehmood et al., 2011), Ca^++^ channel blockade (Gilani et al., 2010; Rehman et al., 2021), phosphodiesterase inhibition (Arshad et al., 2016; Imam et al., 2020) and/or potassium channel activation (Ansari et al., 2024; Khan et al., 2011). Notably, EOCP reversed CCh-induced contractions more effectively than those caused by high K^+^. Dicyclomine, which blocks both muscarinic receptors and calcium (Ca^++^) influx (McGrath et al., 1964; Downie et al., 1977), displayed a similar inhibition pattern, whereas verapamil, a known Ca^++^ signaling inhibitor (Jenkinson, 2002), was observed to be potent for reversing high K^+^-provoked spasms than those induced by CCh. Atropine, a muscarinic receptor non-specific blocker (Arunlakshana and Schild, 1997), only relaxed CCh-induced contractions. These results suggested that EOCP has both muscarinic receptor blocking and Ca^++^ influx inhibitory effects.

Further confirmation of EOCP’s effects was obtained by constructing CCh and Ca^++^ CRCs in the presence of different concentrations of tested samples. At lower doses, EOCP caused a parallel shift in CCh curves without reducing the maximal effect, indicating competitive inhibition, similar to atropine (Eglen and Harris, 1993; Jenkinson, 2002). At higher concentrations, EOCP produced a non-parallel deflection with a reduced peak effect previously observed in control curves, suggesting non-competitive inhibition, as observed with Ca^++^ antagonists (Van den Brink, 1973; Irie et al., 2000). Dicyclomine also deflected the CCh curves in a pattern similar to EOCP, while verapamil caused a rightward, non-parallel manner shift of curves with a reduction in the maximum peaks at all pre-incubated concentrations. In contrast, atropine caused a parallel rightward shift without reducing the maximum response. EOCP shifted the Ca^++^ curves to the right and reduced the maximal response, akin to the effects of verapamil and dicyclomine. Our findings align with the study by Marghich et al. (2023), which demonstrated that the myorelaxant and antispasmodic effects of *Artemisia campestris* essential oil are likely mediated through conformational changes in cholinergic muscarinic receptors and inhibition of L-type voltage-gated calcium channels, with a predominant role attributed to the former mechanism. These findings were further supported by molecular docking analyses. Similarly, several studies on medicinal plants with antidiarrheal and antispasmodic properties (Aleem and Janbaz, 2018; Mehmood et al., 2011) have reported smooth muscle relaxant mechanisms comparable to those observed for EOCP, reinforcing the validity of our current results.

Cholinergic antagonists are well-established therapeutic agents for the treatment of diarrhea (Pietrusko, 1979); however, their use is often associated with adverse cardiac stimulation, particularly when administered orally (Nawarth, 1981). Conversely, calcium channel blockers (CCBs) are also effective in managing hypermotile gut conditions (Borsari, 1991) but are known for their cardio-suppressant effects (Billman, 1992). The presence of CCB-like constituents alongside antimuscarinic compounds in *Cymbopogon proximus* essential oil may represent a naturally occurring mechanism designed to counteract tachycardia, a common side effect of standalone anticholinergic agents. This phenomenon aligns with the concept that natural remedies often exhibit “effect-enhancing and/or side-effect-neutralizing” properties (Gilani and Rahman, 2005), in addition to their cost-effectiveness and potential merit in evidence-based medicine (Ernst, 2005).

This study builds on the traditional use of *Cymbopogon* species as natural remedies to treat gastrointestinal disorders, such as diarrhea and spasms, and supports their historical use in folk medicine (Zhao et al., 2024). For instance, *Cymbopogon citratus* essential oil and its major component, citral, exhibit strong antidiarrheal effects by reducing intestinal motility and mitigating gut hyperactivity (Tangpu and Yadav, 2006). Additionally, *Cymbopogon martinii* essential oil exhibits spasmolytic properties by inhibiting spontaneous contractions in the isolated rabbit jejunum and suppressing potassium-induced contractions, functioning similarly to calcium channel blockers such as verapamil (Janbaz et al., 2014). Research also revealed that *Cymbopogon schoenanthus* essential oil from Sudan has remarkable spasmolytic activity (Pavlović et al., 2017; Djemam et al., 2020).

The effects observed in *C. proximus* may be attributed to the rich composition of its essential oils (Al-Taweel et al., 2013; Althurwi et al., 2020). Notably, some of the identified components in *C. proximus* are known to have spasmolytic properties. For example, Piperitone, a major metabolite in the essential oil studied, was previously shown to have concentration-dependent spasmolytic activity (Ponce-Monter et al., 2008). Additionally, β-eudesmol, another metabolite of *C. Proximus* essential oil, has shown relaxing effect in previous studies (Morita et al., 1996). Limonene, another minor component of this oil, has also been proven to have spasmolytic activity (Cardoso Lima et al., 2012).

This study provides valuable insight into the dual mechanism of *C. proximus* essential oil (EOCP) as an antidiarrheal and antispasmodic agent, but several limitations should be acknowledged. The results based on animal models, may not be directly applicable to humans, and the study lacks examination of other relevant physiological pathways and detailed pharmacokinetic data. Additionally, more research is needed to identify the key components responsible for the observed effects. However, previous studies have documented the antispasmodic effects of *C. Proximus*. This study introduces novel insights by providing a more detailed mechanistic understanding, showing that EOCP acts through both muscarinic receptor inhibition and calcium influx blockade.

## 5 Conclusion

These results suggest that the essential oil extracted from *C. proximus*, independently, had a direct relaxant and spasmolytic effect on the intestinal smooth muscles that does not depend on interaction with receptors for neurotransmitters and are probably myogenic in nature. Moreover, the antidiarrheal and antispasmodic effects of EOCP is to a great extent may be mediated by the dual inhibition of muscarinic receptors and Ca^++^ channels. However, the possible involvement of additional inhibitory mechanism(s) cannot be ruled out. Thus, this study provides a scientific rationale for the traditional use of *C. proximus* in treating gut hyperactivity disorders such as diarrhea and spasms, while paving the way for future studies to explore its potential for broader therapeutic applications. Future research should also examine its long-term safety, effectiveness, and the molecular mechanisms underlying its actions.

## Data Availability

The raw data supporting the conclusions of this article will be made available by the authors, without undue reservation.
